# Supplementary material for: The Association between Fast Food Outlets and Overweight in Adolescents Is Confounded by Neighbourhood Deprivation: A Longitudinal Analysis of the Millennium Cohort Study
Source: Int J Environ Res Public Health. 2021 Dec 15;18(24):13212. doi: 10.3390/ijerph182413212 (PMC8703340; doi:10.3390/ijerph182413212)
Supplement: Supplementary file 1 [file ijerph-18-13212-s001.zip › ijerph-1411565-supplementary.pdf]

Supplementary Appendix

Hypothesis S1: Individuals who live in areas with more fast food outlets have a higher risk of overweight/obesity

Table S1: Results from two Structural Equation Models analysing the association between count of fast food outlets and risk of overweight/obesity in waves 5 and 6 of Millennium Cohort Study.

		Estimate	Lower CI	Upper CI	p value	n
**Model 1: LSOA**						
Outcome	Wave 6 Overweight/obesity					8619
	Wave 6 fast food outlets	0.9927	0.9642	1.0220	0.622	
	Wave 5 Overweight/obesity	31.385	27.551	35.752	<0.001	
Outcome	Wave 5 Overweight/obesity					7101
	Wave 5 fast food outlets	1.0137	0.9851	1.0406	0.310	
Outcome	Wave 6 fast food outlets					7159
	Wave 5 fast food outlets	1.2213	1.2169	1.2257	<0.001	
**Model 2: Local Authority**						
Outcome	Wave 6 Overweight/obesity					8619
	Wave 6 fast food outlets	1.00049	1.00015	1.00083	<0.001	
	Wave 5 Overweight/obesity	31.233	27.416	35.582	<0.001	
Outcome	Wave 5 Overweight/obesity					7101
	Wave 5 fast food outlets	1.00059	1.00023	1.00091	<0.001	
Outcome	Wave 6 fast food outlets					7159
	Wave 5 fast food outlets	1.00322	1.00321	1.00323	<0.001	

Model 1 fit n = 9224, AIC = 36340.46, BIC = 36390.37; Model 2 fit: n = 9224, AIC = 317750.8, BIC 317800.7

Regression models varied by outcome. Overweight/obesity was modelled as binomial logit. Fast food outlets as was modelled using a Poisson regression (selected due to the distribution of the variable which was positively skewed representing a count model distribution). Use of linear models or alternative count models did not alter the findings. Estimate therefore relates to type of model used (odds ratio for binary and ordinal models, incidence rate ratio for count/poisson models).

Hypothesis S2: Individual's who live in deprived areas have a higher risk of overweight/obesity.

Table S2: Results from two Structural Equation Models analysing the association between IMD quintile and risk of overweight/obesity in waves 5 and 6 of Millennium Cohort Study.

		Odds Ratio	Lower CI	Upper CI	p value	n
Outcome	Wave 6 Overweight/obesity					9058
	Wave 6 IMD Quintile 1	1.40565	1.15582	1.70949	0.001	

	Wave 6 IMD Quintile 2	1.36378	1.11046	1.67488	0.003	
	Wave 6 IMD Quintile 3	1.17721	0.95490	1.45129	0.127	
	Wave 6 IMD Quintile 4	1.11518	0.90042	1.38117	0.318	
	Wave 6 IMD Quintile 5	Reference				
	Wave 5 Overweight/Obesity	30.456	26.817	34.589	<0.001	
Outcome	Wave 5 Overweight/obesity					9504
	Wave 5 IMD Quintile 1	1.88991	1.63525	2.18424	<0.001	
	Wave 5 IMD Quintile 2	1.66992	1.43314	1.94583	<0.001	
	Wave 5 IMD Quintile 3	1.58153	1.35507	1.84583	<0.001	
	Wave 5 IMD Quintile 4	1.25060	1.06645	1.46654	0.006	
	Wave 5 IMD Quintile 5	Reference				
Outcome	Wave 6 IMD Quintile					9718
	Wave 5 IMD Quintile 1	1.75e-08	1.17e-08	2.63e-08	<0.001	
	Wave 5 IMD Quintile 2	2.92e-06	2.07e-06	4.11e-06	<0.001	
	Wave 5 IMD Quintile 3	0.000155	0.000115	0.00207	<0.001	
	Wave 5 IMD Quintile 4	0.007499	0.005910	0.00952	<0.001	
	Wave 5 IMD Quintile 5	Reference				

Model fit n=9730AIC = 27432.35, BIC=27568.83.

IMD is modelled as ordinal variable (no different if multinomial), obesity as binomial logit again

Regression models varied by outcome. Neighbourhood deprivation (quintile) was modelled as an ordinal variable. We also tested multinomial regression which produced similar findings. Overweight/obesity was modelled as binomial logit. Estimates are all odds ratios.

Hypothesis S3: Area deprivation confounds the association between fast food outlets and overweight/obesity.

Table S3: Results from two Structural Equation Models analysing the association between count of fast food outlets for Lower Super Output Areas, IMD quintile and risk of overweight/obesity in waves 5 and 6 of Millennium Cohort Study.

		Estimate	Lower CI	Upper CI	p value	n
Outcome	Wave 6 Overweight/obesity					8613
	Wave 6 fast food outlets	0.9807	0.95185	1.0103	0.199	
	Wave 5 Overweight/obesity	30.6741	26.9165	34.9561	<0.001	
	Wave 6 IMD Quintile 1	1.42882	1.1623	1.75645	0.001	
	Wave 6 IMD Quintile 2	1.38343	1.11637	1.71436	0.003	
	Wave 6 IMD Quintile 3	1.17145	0.9422	1.45649	0.154	
	Wave 6 IMD Quintile 4	1.11591	0.89452	1.39208	0.331	
	Wave 6 IMD Quintile 5	Reference				
Outcome	Wave 5 Overweight/obesity					7101
	Wave 5 fast food outlets	0.99002	0.96343	1.01735	0.470	
	Wave 5 IMD Quintile 1	1.94992	1.64083	2.31723	<0.001	

	Wave 5 IMD Quintile 2	1.84176	1.54335	2.19786	<0.001	
	Wave 5 IMD Quintile 3	1.62145	1.35747	1.93675	<0.001	
	Wave 5 IMD Quintile 4	1.31426	1.09772	1.57352	0.003	
	Wave 5 IMD Quintile 5	Reference				
<u>Outcome</u>	<u>Wave 5 Fast food outlets</u>					7151
	Wave 5 IMD Quintile 1	3.03133	2.80113	3.28045	<0.001	
	Wave 5 IMD Quintile 2	2.66304	2.45445	2.88935	<0.001	
	Wave 5 IMD Quintile 3	1.82734	1.67536	1.99311	<0.001	
	Wave 5 IMD Quintile 4	1.50715	1.37799	1.64841	<0.001	
	Wave 5 IMD Quintile 5	Reference				
<u>Outcome</u>	<u>Wave 6 Fast food outlets</u>					7269
	Wave 6 IMD Quintile 1	2.10174	1.94664	2.692	<0.001	
	Wave 6 IMD Quintile 2	2.00574	1.85393	2.16999	<0.001	
	Wave 6 IMD Quintile 3	1.64206	1.51272	1.78245	<0.001	
	Wave 6 IMD Quintile 4	1.36933	1.25735	1.49128	<0.001	
	Wave 6 IMD Quintile 5	Reference				
	Wave 5 fast food outlets	1.20655		1.201975	1.211143	<0.001
<u>Outcome</u>	<u>Wave 6 IMD Quintile</u>					9718
	Wave 5 IMD Quintile 1	1.75e-8		1.17e-08	2.63e-08	<0.001
	Wave 5 IMD Quintile 2	2.93e-06		2.07e-06	4.11e-06	<0.001
	Wave 5 IMD Quintile 3	0.0001545		0.000115	0.00021	<0.001
	Wave 5 IMD Quintile 4	0.007499		0.00591	0.009515	<0.001
	Wave 5 IMD Quintile 5	Reference				

Model fit n=9729, AIC = 70879.11, BIC = 71108.96

Table S4: Results from two Structural Equation Models analysing the association between count of fast food outlets for Local Authorities, IMD quintile and risk of overweight/obesity in waves 5 and 6 of Millennium Cohort Study.

		**Estimate**	**Lower CI**	**Upper CI**	**p value**	**n**
<u>Outcome</u>	<u>Wave 6 Overweight/obesity</u>					8613
	Wave 6 fast food outlets	1.00027	0.99989	1.00066	0.162	
	Wave 5 Overweight/obesity	30.7162	26.9523	35.0056	<0.001	
	Wave 6 IMD Quintile 1	1.30864	1.04884	1.63278	0.017	
	Wave 6 IMD Quintile 2	1.31831	1.06089	1.63819	0.013	
	Wave 6 IMD Quintile 3	1.1384	0.91482	1.41662	0.245	
	Wave 6 IMD Quintile 4	1.09648	0.878447	1.36863	0.415	
	Wave 6 IMD Quintile 5	Reference				
<u>Outcome</u>	<u>Wave 5 Overweight/obesity</u>					7101
	Wave 5 fast food outlets	1.0001	0.99975	1.00046	0.578	
	Wave 5 IMD Quintile 1	1.8916	1.57523	2.27144	<0.001	
	Wave 5 IMD Quintile 2	1.80876	1.51367	2.16136	<0.001	
	Wave 5 IMD Quintile 3	1.60502	1.34279	1.91846	<0.001	
	Wave 5 IMD Quintile 4	1.30517	1.0896	1.56334	<0.001	
	Wave 5 IMD Quintile 5	Reference				
<u>Outcome</u>	<u>Wave 5 Fast food outlets</u>					7269

	Wave 5 IMD Quintile 1	2.87045	2.85378	2.88721	<0.001	
	Wave 5 IMD Quintile 2	1.90054	1.8886	1.91256	<0.001	
	Wave 5 IMD Quintile 3	1.54127	1.53118	1.55143	<0.001	
	Wave 5 IMD Quintile 4	1.41461	1.40524	1.42405	<0.001	
	Wave 5 IMD Quintile 5	Reference				
<u>Outcome</u>	<u>Wave 6 Fast food outlets</u>					7151
	Wave 6 IMD Quintile 1	1.3402	1.33224	1.34821	<0.001	
	Wave 6 IMD Quintile 2	1.28188	1.27401	1.28979	<0.001	
	Wave 6 IMD Quintile 3	1.18506	1.17757	1.19261	<0.001	
	Wave 6 IMD Quintile 4	1.12679	1.11962	1.133999	<0.001	
	Wave 6 IMD Quintile 5	Reference				
	Wave 5 fast food outlets	1.003064		1.003057	1.003072	<0.001
<u>Outcome</u>	<u>Wave 6 IMD Quintile</u>					9718
	Wave 5 IMD Quintile 1	1/75e-08		1.17e-08	2.63e-08	<0.001
	Wave 5 IMD Quintile 2	2.92e-06		2.07e-06	4.11e-06	<0.001
	Wave 5 IMD Quintile 3	0.001545		0.000115	0.000207	<0.001
	Wave 5 IMD Quintile 4	0.007499		0.00591	0.009515	<0.001
	Wave 5 IMD Quintile 5	Reference				

Model fit n=9729, AIC=1067989, BIC=1068219

In both Tables A3 and A4, overweight was modelled using a logistic regression model, IMD quintile was modelled using an ordinal variable, count of takeaway was modelled using a count (poisson) regression model (this was due to evidence of positive skew in the data). We tested the use of a linear regression model for both LSOAs and LADs, however they resulted in poorer fit and did not alter the associations detected. Estimate therefore relates to type of model used (odds ratio for binary and ordinal models, incidence rate ratio for count/poisson models).

We repeated the same analyses as described above but changing the exposures for fast food outlets to:

- Count per population
- Proportion of total outlets that are fast food outlets
- Ratio of fast food outlets to supermarkets
- Convert food environment counts to tertiles (quintiles were not possible for LSOA values)

Results are presented below:

Table S5: Results from two Structural Equation Models (one at LSOA level and one at LAD level) analysing the association between measures of the food environment and risk of overweight/obesity in waves 5 and 6 of Millennium Cohort Study (adjusting for deprivation).

Scale	Measure	Odds Ratio	Lower CI	Upper CI	p value
	<u>Proportion of all outlets as fast food outlets</u>				
LSOAs	Wave 6 takeaway	0.92621	0.81149	1.05715	0.256
	Wave 5 takeaway	1.00098	0.82395	1.21605	0.992
LADs	Wave 6 takeaway	0.99275	0.97441	1.01144	0.445

	Wave 5 takeaway	1.83362	1.09204	3.07881	0.022
	Ratio of fast food outlets to supermarkets				
LSOAs	Wave 6 ratio	0.950647	0.881996	1.02464	0.186
	Wave 5 ratio	1.00092	0.94794	1.05687	0.974
LADs	Wave 6 ratio	0.99388	0.96959	1.01879	0.627
	Wave 5 ratio	0.998575	0.99196	1.00524	0.674
	Tertiles				
LSOAs	Wave 6 tertile 1 (least)	Reference			
	Wave 6 tertile 2	0.76007	0.63409	0.91108	0.003
	Wave 6 tertile 3 (Most)	0.86848	0.74368	1.01422	0.075
	Wave 5 tertile 1 (least)	Reference			
	Wave 5 tertile 2	0.921586	0.79674	1.06599	0.272
	Wave 5 tertile 3 (Most)	0.99737	0.87692	1.13437	0.968
LADs	Wave 6 tertile 1 (least)	Reference			
	Wave 6 tertile 2	0.95516	0.800338	1.13993	0.611
	Wave 6 tertile 3 (Most)	0.874295	0.74404	1.02735	0.103
	Wave 5 tertile 1 (least)	Reference			
	Wave 5 tertile 2	0.92295	0.80224	1.06182	0.262
	Wave 5 tertile 3 (Most)	0.96321	0.84030	1.10400	0.590
	Count per pop				
LSOAs	Wave 6 rate	1.10e-16	5.35e-40	2.25e+07	0.180
	Wave 5 rate	0.000048	8.95e-25	2.61e+15	0.668
LADs	Wave 6 rate	4.00e-47	3.5e-152	4.55e+58	0.387
	Wave 5 rate	3.94e+70	7.60e-09	2.0e+149	0.079

Note: Wave 5 n = 7101, Wave 6 n = 8613.

In all models, logistic regression was used to model overweight.

Hypothesis S4: Areas where the number of fast food outlets increased, individuals had higher likelihood of being overweight/obese.

Table S6: Results from two Structural Equation Models (one at LSOA level and one at LAD level) examining the association between change in the number of takeways between waves 5 and 6 as predictor of and risk of overweight (adjusting for deprivation).

		Estimate	Lower CI	Upper CI	p value	n
**Model 6: LSOA**						6784
Outcome	Wave 6 Overweight/obesity					
	Change in count	0.97846	0.94594	1.0121	0.207	
	IMD Q1	2.15152	1.8074	2.56116	<0.001	
	IMD Q2	1.85215	1.5469	2.2175	<0.001	
	IMD Q3	1.52537	1.2692	1.8332	<0.001	
	IMD Q4	1.17445	0.97321	1.4173	0.094	
	IMD Q5	Reference				

Outcome	Change in count					7151
	IMD Q1	0.22794	0.11265	0.34322	<0.001	
	IMD Q2	0.214477	0.09594	0.33301	<0.001	
	IMD Q3	0.05708	-0.06195	0.17612	0.347	
	IMD Q4	0.02163	-0.09558	0.13884	0.718	
	IMD Q5	Reference				
**Model 7: Local Authority**						6784
Outcome	Wave 6 Overweight/obesity					
	Change in count	1.00041	0.99964	1.00118	0.3	
	IMD Q1	2.1327	1.7916	2.5388	<0.001	
	IMD Q2	1.83357	1.53116	2.1957	<0.001	
	IMD Q3	1.5212	1.2657	1.8282	<0.001	
	IMD Q4	1.1746	0.9733	1.4175	0.093	
	IMD Q5	Reference				
Outcome	Change in count					7151
	IMD Q1	9.15678	4.2785	14.035	<0.001	
	IMD Q2	13.3319	8.3159	18.3479	<0.001	
	IMD Q3	4.536	-0.5010	9.573	0.078	
	IMD Q4	-0.5227	-5.4825	4.437	0.836	
	IMD Q5	Reference				

*No association between Quintiles and LSOA change

Logistic regression was used to model overweight. Where the change in count was the outcome variable, we used a linear OLS regression model as the outcome was normally distributed (converting the data to a categorical variable for ‘increased’, ‘no change’ and ‘decreased’ produced similar results and had poorer model fit. Estimate therefore relates to type of model used (odds ratio for binary and ordinal models, beta for linear models).

Hypothesis S5: Including physical activity and diet in the model does not change the core findings.

Modelling here follows the same as described in hypothesis 3. For both fast food consumption and physical activity, we modelled each using ordinal regression models.

Table S7: Results from Structural Equation Models analysing the determinants of overweight in waves 5 and 6 of Millennium Cohort Study (LSOA data for fast food outlets).

		**estimate**	**Lower CI**	**Upper CI**	**p value**	**n**
Outcome	Wave 6 Fast food outlets					7151
	Wave 5 fast food outlets	1.2066	1.202	1.2111	<0.001	
	Wave 6 IMD Quintile 1	2.1017	1.9466	2.2692	<0.001	

	Wave 6 IMD Quintile 2	2.0057	1.8539	2.1699	<0.001	
	Wave 6 IMD Quintile 3	1.6421	1.5127	1.78245	<0.001	
	Wave 6 IMD Quintile 4	1.3633	1.2574	1.4913	<0.001	
	Wave 6 IMD Quintile 5	Reference				
Outcome	Wave 5 Overweight/obesity					7101
	Wave 5 fast food outlets	0.99002	0.96343	1.01734	0.47	
	Wave 5 IMD Quintile 1	1.9499	1.6408	2.3172	<0.001	
	Wave 5 IMD Quintile 2	1.8418	1.5434	2.1979	<0.001	
	Wave 5 IMD Quintile 3	1.6214	1.3575	1.9368	<0.001	
	Wave 5 IMD Quintile 4	1.3143	1.0977	1.5735	0.003	
	Wave 5 IMD Quintile 5	Reference				
Outcome	Days MVPA					9309
	Wave 5 overweight/obese	1.5065	1.3849	1.6388	<0.001	
	Wave 6 IMD Quintile 1	1.3952	1.2473	1.5607	<0.001	
	Wave 6 IMD Quintile 2	1.4023	1.2468	1.5772	<0.001	
	Wave 6 IMD Quintile 3	1.4154	1.2583	1.592	<0.001	
	Wave 6 IMD Quintile 4	1.3227	1.1779	1.4852	<0.001	
	Wave 6 IMD Quintile 5	Reference				
Outcome	Wave 6 Fast food					8824
	Wave 6 fast food outlets	0.984	0.9661	1.0022	0.085	
	Wave 5 overweight/obesity	0.7922	0.7245	0.8662	<0.001	
	Wave 6 IMD Quintile 1	3.9887	3.5179	4.52249	<0.001	
	Wave 6 IMD Quintile 2	2.5814	2.2716	2.9334	<0.001	
	Wave 6 IMD Quintile 3	1.852	1.6314	2.1025	<0.001	
	Wave 6 IMD Quintile 4	1.2946	1.1423	1.1467	<0.001	
	Wave 6 IMD Quintile 5	Reference				
Outcome	Wave 5 Fast food outlets					7269
	Wave 5 IMD Quintile 1	3.0313	2.8011	3.2804	<0.001	
	Wave 5 IMD Quintile 2	2.663	2.45445	2.8893	<0.001	
	Wave 5 IMD Quintile 3	1.8273	1.6754	1.9931	<0.001	
	Wave 5 IMD Quintile 4	1.5071	1.378	1.6484	<0.001	
	Wave 5 IMD Quintile 5	Reference				
Outcome	Wave 6 Overweight/obesity					8535
	Wave 6 fast food Less/never	Reference				
	Monthly	0.9434	0.80549	1.105	0.47	
	Weekly	0.8084	0.674	0.9695	0.022	
	Days MVPA Every day	Reference				
	5-6 days	1.369	1.0839	1.72998	0.008	
	3-4 days	1.610	1.3108	1.9777	<0.001	
	2 or less days	2.0387	1.6552	2.511	<0.001	
	Wave 5 overweight/obese	29.739	26.06	33.937	<0.001	
	Wave 6 IMD Quintile 1	1.4609	1.1791	1.81	0.001	
	Wave 6 IMD Quintile 2	1.4104	1.1333	1.7553	0.002	
	Wave 6 IMD Quintile 3	1.1373	0.91178	1.4186	0.254	
	Wave 6 IMD Quintile 4	1.0876	0.8702	1.3592	0.46	
	Wave 6 IMD Quintile 5	Reference				

	Wave 6 fast food outlets	0.9748		0.9459	1.0022	0.085
Outcome	Wave 6 IMD Quintile					9718
	Wave 5 IMD Quintile 1	1.75e-08		1.17e-08	2.63e-08	<0.001
	Wave 5 IMD Quintile 2	2.92e-06		2.07e-06	4.11e-06	<0.001
	Wave 5 IMD Quintile 3	0.0001545		0.000151	0.000207	<0.001
	Wave 5 IMD Quintile 4	0.0075		0.0059	0.00952	<0.001
	Wave 5 IMD Quintile 5	Reference				

Model fit n=9729, AIC = 114123.8, BIC = 114505.5

Table S8: Results from Structural Equation Models analysing the determinants of overweight in waves 5 and 6 of Millennium Cohort Study (LAD data for fast food outlets).

		estimate	Lower CI	Upper CI	p value	n
**Model 10: LADs**						
Outcome	Wave 6 Fast food outlets					7151
	Wave 5 fast food outlets	1.00306	1.003057	1.00307	<0.001	
	Wave 6 IMD Quintile 1	1.3402	1.33222	1.3482	<0.001	
	Wave 6 IMD Quintile 2	1.2819	1.274	1.2898	<0.001	
	Wave 6 IMD Quintile 3	1.1851	1.1776	1.1926	<0.001	
	Wave 6 IMD Quintile 4	1.1268	1.1196	1.1339	<0.001	
	Wave 6 IMD Quintile 5	Reference				
Outcome	Wave 5 Overweight/obesity					7101
	Wave 5 fast food outlets	1.0001	0.9997	1.0004	0.578	
	Wave 5 IMD Quintile 1	1.8916	1.5772	2.2714	<0.001	
	Wave 5 IMD Quintile 2	1.8088	1.5137	2.1614	<0.001	
	Wave 5 IMD Quintile 3	1.605	1.3428	1.91846	<0.001	
	Wave 5 IMD Quintile 4	1.3052	1.0896	1.56339	0.004	
	Wave 5 IMD Quintile 5	Reference				
Outcome	Days MVPA					9309
	Wave 5 overweight/obese	1.5065	1.3894	1.6388	<0.001	
	Wave 6 IMD Quintile 1	1.3952	1.2473	1.5607	<0.001	
	Wave 6 IMD Quintile 2	1.4023	1.2468	1.5772	<0.001	
	Wave 6 IMD Quintile 3	1.4154	1.2583	1.592	<0.001	
	Wave 6 IMD Quintile 4	1.3227	1.1779	1.4852	<0.001	
	Wave 6 IMD Quintile 5	Reference				
Outcome	Wave 6 Fast food					8824
	Wave 6 fast food outlets	1.0008	1.0005	1.001	<0.001	
	Wave 5 overweight/obesity	0.7892	0.7217	0.8629	<0.001	
	Wave 6 IMD Quintile 1	3.298	2.8846	3.7707	<0.001	
	Wave 6 IMD Quintile 2	2.3443	2.0603	2.6675	<0.001	
	Wave 6 IMD Quintile 3	1.7469	1.5379	1.9844	<0.001	
	Wave 6 IMD Quintile 4	1.243	1.0963	1.4094	0.001	
	Wave 6 IMD Quintile 5	Reference				
Outcome	Wave 5 Fast food outlets					7269
	Wave 5 IMD Quintile 1	2.8704	2.8538	2.8872	<0.001	
	Wave 5 IMD Quintile 2	1.9005	1.8886	1.9126	<0.001	

	Wave 5 IMD Quintile 3	1.5413	1.5312	1.5514	<0.001	
	Wave 5 IMD Quintile 4	1.4146	1.4052	1.4240	<0.001	
	Wave 5 IMD Quintile 5	Reference				
Outcome	Wave 6 Overweight/obesity					8535
	Wave 6 fast food Less/never	Reference				
	Monthly	0.9434	0.8055	1.1049	0.47	
	Weekly	0.8037	0.67	0.9642	0.019	
	Days MVPA Every day	Reference				
	5-6 days	1.3653	1.0805	1.7251	0.009	
	3-4 days	1.6107	1.3113	1.9784	<0.001	
	2 or less days	2.0363	1.6531	2.5084	<0.001	
	Wave 5 overweight/obese	29.783	26.098	33.989	<0.001	
	Wave 6 IMD Quintile 1	1.3042	1.0389	1.6373	0.022	
	Wave 6 IMD Quintile 2	1.3261	1.0631	1.654	0.012	
	Wave 6 IMD Quintile 3	1.0966	0.8785	1.3689	0.415	
	Wave 6 IMD Quintile 4	1.0635	0.8506	1.3298	0.589	
	Wave 6 IMD Quintile 5	Reference				
	Wave 6 fast food outlets	1.0004		0.9999	1.0007	0.069
Outcome	Wave 6 IMD Quintile					9718
	Wave 5 IMD Quintile 1	1.75e-08		1.17e-08	2.63e-08	<0.001
	Wave 5 IMD Quintile 2	2.92e-06		2.07e-06	4.11e-06	<0.001
	Wave 5 IMD Quintile 3	0.00015		0.00012	0.00021	<0.001
	Wave 5 IMD Quintile 4	0.0075		0.0059	0.0095	<0.001
	Wave 5 IMD Quintile 5	Reference				

Model fit n = 9729, AIC = 1111198, BIC = 1111579.

Estimate relates to type of model used (odds ratio for binary and ordinal models, incidence rate ratio for count/poisson models).

Additional sensitivity analysis on attrition

Finally, we describe the extent of attrition. Of the wave 5 participants who dropped out before wave 6, 34% were in the most deprived quintile compared to 14% in the least deprived quintile. The uneven split of attrition by deprivation limits our findings. There was less bias observed by measures of fast food outlets. For example, 38% of participants who dropped out where from the highest tertile of fast food outlets for LADs compared to 32% in the lowest.
